# Postabortion and safe abortion care coverage, capacity, and caseloads during the global gag rule policy period in Ethiopia and Uganda

**DOI:** 10.1186/s12913-022-09017-8

**Published:** 2023-02-01

**Authors:** Melissa Stillman, Simon P. S. Kibira, Solomon Shiferaw, Fredrick Makumbi, Assefa Seme, Elizabeth A. Sully, Lilian Han, Margaret Giorgio

**Affiliations:** 1 Guttmacher Institute, 125 Maiden Lane, New York, NY 10038 USA; 2 School of Public Health, College of Health Sciences, Makerere University, New Mulago Hill Rd, Kampala, Uganda; 3 School of Public Health, Addis Ababa University, Addis Ababa, Ethiopia

**Keywords:** Postabortion care, Safe abortion, Quality, Signal functions, Global gag rule

## Abstract

**Background:**

Abortion-related complications contribute to preventable maternal mortality, accounting for 9.8% of maternal deaths globally, and 15.6% in sub-Saharan Africa. High-quality postabortion care (PAC) can mitigate the negative health outcomes associated with unsafe abortion. While the expanded Global Gag Rule policy did not prohibit the provision of PAC, other research has suggested that over-implementation of the policy has resulted in impacts on these services. The purpose of this study was to assess health facilities’ capacity to provide PAC services in Uganda and PAC and safe abortion care (SAC) in Ethiopia during the time in which the policy was in effect.

**Methods:**

We collected abortion care data between 2018 and 2020 from public health facilities in Ethiopia (*N* = 282) and Uganda (*N* = 223). We adapted a signal functions approach to create composite indicators of health facilities’ capacity to provide basic and comprehensive PAC and SAC and present descriptive statistics documenting the state of service provision both before and after the GGR went into effect. We also investigate trends in caseloads over the time-period.

**Results:**

In both countries, service coverage was high and improved over time, but facilities’ capacity to provide basic PAC services was low in Uganda (17.8% in 2019) and Ethiopia (15.0% in 2020). The number of PAC cases increased by 15.5% over time in Uganda and decreased by 7% in Ethiopia. Basic SAC capacity increased substantially in Ethiopia from 66.7 to 82.8% overall, due in part to an increase in the provision of medication abortion, and the number of safe abortions increased in Ethiopia by 9.7%.

**Conclusions:**

The findings from this analysis suggest that public health systems in both Ethiopia and Uganda were able to maintain essential PAC/SAC services during the GGR period. In Ethiopia, there were improvements in the availability of safe abortion services and an overall improvement in the safety of abortion during this time-period. Despite loss of partnerships and potential disruptions in referral chains, lower-level facilities were able to expand their capacity to provide PAC services. However, PAC caseloads increased in Uganda which could indicate that, as hypothesized, abortion became more stigmatized, less accessible and less safe.

## Background

Safe abortion services are an essential component of sexual and reproductive healthcare [1]. Yet, it is estimated that 45% of abortions occurring annually around the world, and 75% of abortions in Africa, are unsafe [2]. Abortion-related complications contribute to preventable maternal mortality and morbidity, accounting for 9.8% of maternal deaths globally, and 15.6% of maternal deaths in sub-Saharan Africa [3]. Timely and high-quality postabortion care (PAC), which is a set of services provided to women who present with complications from unsafe or incomplete abortions, can greatly mitigate the negative health outcomes associated with unsafe abortion. While all countries have committed to providing PAC to reduce the burden of abortion-related morbidity and mortality, [4] sufficient health system capacity to provide PAC is not universal [5].

Abortion care was targeted as part of the Trump Administration’s Protecting Life in Global Health Assistance policy, also known as the Global Gag Rule (GGR) [6]. This iteration of the GGR prohibited non-U.S. non-government organizations (NGOs) and their subgrantees that receive U.S. government global health assistance from using their own non-U.S. funds to provide, refer to, or advocate for safe abortion care (SAC) services. Despite this seemingly narrow focus, there is some evidence to suggest that the policy affected other areas of sexual and reproductive health (SRH): previous quantitative research assessing the impacts of the GGR has documented impacts on the delivery of family planning services in both the public and private sector, [7–9] subsequent impacts on women’s SRH outcomes that are likely a result of changes in family planning service delivery, [8, 10–12] and changes in the delivery of HIV services [13].

Despite the increasing evidence of the negative impact of the GGR on several SRH outcomes, little is known about the GGR’s impact on the facility-based provision of PAC or SAC itself. While the policy does not prohibit the provision of PAC, some evidence suggests the existence of “chilling effects”, where providers avoid even permitted services due to fears of withdrawal or loss of funding, has resulted in impacts on these services [14]. Further, implementation has likely affected access to abortion services even in settings where abortion is widely available, regardless of abortion legality, by disrupting health system partnerships, referral mechanisms, and access to information and safe services.

Shedding light on the GGR’s real-world effects on the availability, quality, and utilization of PAC and SAC services is important for informing the design of policies and programs aimed at reducing maternal morbidity and mortality. In this paper, we compare health systems’ capacities to provide SAC and/or PAC, as well as trends in these indicators while the GGR was in effect for two countries: Ethiopia and Uganda. Both countries are uniquely susceptible to changes in U.S. global health funding policies. The U.S. is the largest donor of global health funding to both Ethiopia and Uganda, and the second-largest donor for Ethiopia’s family planning budget [15, 16]. Since the expansion of the abortion law in Ethiopia in 2005, there have been significant increases in the availability and quality of abortion services. Between 2008 and 2014, use of appropriate technology for conducting first and second trimester abortions, including medication abortion, as well as provision of postabortion family planning, has increased, while abortion-related complications have decreased [17–19]. However, regional disparities persist, and NGOs play a critical role in delivering high quality SRH services, both directly and through public-private partnerships. In Uganda, abortion remains highly restricted, prohibited under all circumstances unless the woman’s life is at risk [20]. Given the links between abortion restrictions and safety, PAC is especially critical in Uganda [2]. To this point, a recent study of maternal near-miss and abortion complications data from health facilities in Central and Eastern Uganda in 2016–2017 found a high burden of abortion-related morbidity and mortality [21].

One way to measure health facilities’ capacity to provide services is through a signal functions approach, originally developed by the United Nations to assess the provision of emergency obstetric care (EmOC) [22]. The methodology typically consists of a list of indicators used to assess health facilities’ capability to provide the most effective or life-saving interventions for managing the most common complications. In recent years, the signal functions approach has been adapted to measure capacity to provide basic and comprehensive SAC as well as PAC, [23, 24] and this framework has since been applied to assess service provision across various geographical contexts [5, 17, 25–28].

In this study, we use a signal functions approach to assess the capacity of facilities to provide PAC services in Uganda and PAC/SAC services in Ethiopia at two points in time while the GGR was in effect. We investigate if and how these services changed during this period and document overall gaps in the capacity of facilities to provide essential services, which is one dimension of quality of care. The results of this study provide insight into how abortion care service delivery in Uganda and Ethiopia was impacted during the years in which the GGR was in effect as well as help identify gaps in service provision that can be addressed to improve capacity within the health systems to prevent and treat abortion-related complications.

## Methods

### Data sources and sample

This analysis utilizes data from multiple health facility surveys in Uganda and Ethiopia. Data in Uganda come from the 2018 and 2019 round of the Performance Monitoring for Action (PMA) platform [29]. In 2018, the PMA platform included a nationally representative survey of women, which used a two-stage cluster sampling design to select a collection of 110 enumeration areas (EAs) [30]. The health facility survey is not nationally representative, but it was designed to be representative of health facilities that provide family planning services to women in the female questionnaires. All public facilities that serve the population in the selected EAs were sampled, regardless of whether that facility was located within the EA. All private facilities within the EA were mapped/listed and up to three were randomly selected. In 2018, a total of 361 health facilities were interviewed. In 2019, study staff attempted to reinterview all facilities in the 2018 sample, and 333 (92.2%) were successfully reinterviewed.

The Ethiopia 2018 health facility data also come from the PMA platform [31]. The sampling procedure in Ethiopia was similar to in Uganda, in that a nationally representative sample of EAs was drawn (*n* = 221), and the sample of health facilities was selected to be representative of those that serve the population in the sampled EAs [32]. The 2020 health facility survey was conducted as part of a larger panel study investigating the impact of the Global Gag Rule [9]. In this data collection effort, all 2018 PMA facilities that were located in six study regions (Addis Ababa, Afar, Amhara, Oromia, SNNPR, Tigray) were eligible to be resurveyed (*n* = 425), and 410 (96.5%) were reinterviewed. Because the sample of private facilities in the baseline PMA surveys was small and non-representative and because many of the private facilities were either pharmacies or drug shops, which are not capable of providing PAC, we excluded private facilities from this analysis. In Ethiopia, Health Posts were also excluded from the analysis because they are not expected to provide either PAC or SAC services. As such, our final analytical sample included 223 public facilities in Uganda and 282 public facilities in Ethiopia.

Surveys were conducted face-to-face by trained enumerators on Android smartphones using Open Data Kit (ODK) software. Informed consent was obtained from all respondents prior to each interview. Ethical approval was provided by the Institutional Review Boards of the Guttmacher Institute, Johns Hopkins Bloomberg School of Public Health, Makerere University, and Addis Ababa University, as well as the Uganda National Council for Science and Technology.

### Measures

#### Facility characteristics

Informed by each country’s national reproductive health guidelines, we categorized facilities as primary- or referral-level. In Uganda, hospitals and level IV health centers were considered referral-level facilities, and level II and III health centers were primary level [33]. In Ethiopia, hospitals were classified as referral-level facilities and health centers as primary-level facilities [34]. We also classified facilities as being located in an urban or rural location. All facilities were asked if they provided PAC. Due to the restrictive abortion law in Uganda, SAC questions were only asked for facilities in Ethiopia.

#### Signal functions

We adapted previous signal functions approaches to create composite indicators of health facilities’ capacity to provide basic and comprehensive PAC (in Uganda and Ethiopia) and SAC (in Ethiopia only) [23, 24]. Relevant signal functions include those related to the availability of specific services, staffing, the provision of postabortion contraception, and structural capacity of facilities (detailed in Table 1). Basic care is defined as the minimum service that primary (and higher) facilities should be able to provide, and comprehensive care is defined as the minimum care that would be expected at referral-level facilities. Our choice of indicators for this study was based on WHO’s technical guidance regarding supplies and human resources for health systems providing abortion care [35]. Some signal functions used in other studies were not collected in the PMA surveys, including facilities being open 24/7, having 3+ health professionals or medical doctors *registered* to provide PAC or SAC (needed for 24/7 services), communications means or referral capacity, and availability of a vehicle with fuel (for facilities without comprehensive PAC).

**Table 1:** Signal functions used to classify postabortion care (PAC) and safe abortion care (SAC) capability

	PAC Capability		SAC Capability	
	Basic	Comprehensive	Basic	Comprehensive
Perform removal of retained products^a^	X	X		
Perform manual vacuum aspiration or electric vacuum aspiration			X	X
Provide medication abortion using mifepristone + misoprostol or misoprostol alone			X	X
Administer parenteral antibiotics^b^	X	X		
Administer uterotonics^b^	X	X		
Administer intravenous fluids^b^	X	X		
Provide at least one short-acting contraceptive (condoms, pills or injectables)^c^	X	X	X	X
Provide at least one long-acting reversible contraceptive (IUDs or implants)^d^		X		X
Offer family planning at least once per week			X	X
Offer family planning 7 days a week	X	X		
Perform blood transfusion^b^		X		
Surgical/laparotomy capability^b^		X		
Perform dilation and evacuation				X
Has 1+ staff capable of providing PAC available^e^	X	X		
Has 1+ staff capable of providing SAC available^f^			X	X

^a^Includes manual/electric vacuum aspiration (MVA/EVA), or misoprostol alone or mifepristone + misoprostol. For MVA/EVA, must have functional equipment at the time of the survey. For mifepristone/misoprostol, medication must be in stock at the time of the survey

^b^Based on facility reporting that they provide the service

^c^Based on at least one method being in stock at the time of the survey

^d^Based on at least one method being in stock at the time of the survey and availability of trained staff

^e^In Ethiopia – Doctors, nurse/midwives, health officers, health extension workers. In Uganda – Doctors, nurse/midwives, clinical officers

^f^In Ethiopia – Doctors, nurse/midwives, or health officers

Facilities’ ability to provide manual/electric vacuum aspiration was assessed based on the respondent reporting that the facility provided it, the availability of trained staff, and the functionality of the equipment. Ability to provide misoprostol, mifepristone and at least one short-acting contraceptive method was assessed based on the medicine/method being in stock at the time of the survey. Ability to provide a long-acting reversible contraceptive method was assessed on the availability of the method at the time of the survey and staff trained to provide at least one method. All other criteria were assessed solely through respondent reporting.

While the definitions for basic and comprehensive PAC and SAC are similar, there is one notable difference: in order for a facility to provide either basic or comprehensive PAC per our definition, it must offer family planning services 7 days a week. Family planning services should be offered to all people at the time they receive abortion or postabortion services. Questions were not asked specifically about when family planning was provided in relation to PAC/SAC services, therefore, we use the number of days family planning is offered as a best approximation with the data available to determine if facilities are able to provide postabortion family planning. Since PAC services may be needed at any time on an emergency basis, for a facility to provide basic or comprehensive PAC services family planning must be available on all days [24]. However, because in most cases SAC may be a scheduled service and is not typically provided on an emergency basis, facilities can theoretically schedule SAC to occur on days when family planning services are available. As such, definitions for basic and comprehensive SAC are less strict and only require a facility to offer family planning 1 day per week.

#### PAC and SAC caseloads

Facility records are generally considered underestimates for documenting the number of PAC and/or SAC cases due to incomplete reporting, regardless of the legal status of abortion [36]. Therefore, we included questions that have been widely used in a well-known methodology for estimating the magnitude of abortion and PAC caseloads [37]. We asked respondents how many PAC patients their facility had treated in the past month and in an average month, which we then averaged and multiplied by 12 to estimate the number of cases treated in the past year. We also measured whether facilities provided inpatient services, referred patients elsewhere, accepted referral patients in the past month, or treated any severe complications in the past month, including perforated uterus or gut requiring laparotomy, intensive care unit admission, or organ failure.

In Ethiopia, we measure annual SAC caseloads using the same method as we did for PAC. We also assessed whether the facility had provided any second trimester abortions in the past month.

### Analysis

First, we describe the proportion of facilities that provide PAC and/or SAC, according to facility type and urban or rural location for both rounds of surveys. Among facilities that provide PAC, we calculated the proportion that met each set of signal functions in the composite indicators to measure the proportion of primary-level and referral-level facilities capable of providing basic PAC, and the proportion of referral-level facilities capable of providing comprehensive PAC services (see Table 1). We also calculated the proportion of facilities that provided/had each individual signal function in order to identify gaps in service capability and provision. In Ethiopia, the same approach was taken to calculate basic and comprehensive SAC.

We also investigate trends in caseloads while the GGR was in effect. In both countries, we present the number of PAC patients in the past year, as well as the average number of cases per facility across both years of surveys. We describe the proportion of facilities that provided inpatient services in the past month, the proportion that either received referrals or referred patients elsewhere, and the proportion that treated severe complications, separately by PAC capability (less than basic/basic/comprehensive), and overall. In Ethiopia, we also present the caseloads for safe abortion, the proportion of facilities that provided second trimester abortion in the past month, and the proportion that had accepted abortion referrals. Consistent with the PAC data, we present these proportions overall and by capability across the two rounds of surveys.

For all measures, we calculate the proportions for the first and second survey rounds separately, the percent difference between the two rounds, and we tested the statistical significance of the differences in the two proportions. All analyses were conducted using Stata 16.0.

## Results

### Availability of postabortion and safe abortion care services in facilities

In Ethiopia, 95.4% of public facilities reported providing either postabortion care or safe abortion services in 2020 (Table 2). All hospitals and 92.9% of health centers provided either service. PAC provision remained consistently high across facility types and locations between 2018 and 2020, while safe abortion provision increased from 76.6 to 84.4% (*p* < 0.02) over the 2 years. Safe abortion services increased most notably among health centers and facilities in rural areas (9.8 and 12.2 percentage point increases, respectively; *p* < 0.03).

**Table 2:** Postabortion care and induced abortion service provision among public facilities, Ethiopia 2018 & 2020 and Uganda 2018 & 2019

	**Ethiopia**																
	2018							2020							% Change 2018–2020		
	Facilities in the sample	Offer postabortion care		Offer induced abortion		Offer either postabortion care or induced abortion		Facilities in the sample	Offer postabortion care		Offer induced abortion		Offer either postabortion care or induced abortion		Offer PAC	Offer SAC	Offer either PAC or SAC
	N	N	%	N	%	N	%	N	N	%	N	%	N	%	%	%	%
All Public facilities	282	259	91.8	216	76.6	262	92.9	282	267	94.7	238	84.4	269	95.4	2.8	7.8*	2.5
Facility type																	
Public Hospital	98	98	100.0	91	92.9	98	100.0	98	98	100.0	95	96.9	98	100.0	0.0	4.1	0.0
Public Health center	184	161	87.5	125	67.9	164	89.1	184	169	91.8	143	77.7	171	92.9	4.3	9.8*	3.8
Location																	
Urban	150	144	96.0	132	88.0	146	97.3	150	146	97.3	138	92.0	148	98.7	1.3	4.0	1.3
Rural	132	115	87.1	84	63.6	116	87.9	132	121	91.7	100	75.8	121	91.7	4.5	12.1	3.8
	**Uganda**																
	2018							2019							% Change 2018–2019		
	Facilities in the sample	Offer postabortion care		Offer induced abortion		Offer either postabortion care or induced abortion		Facilities in the sample	Offer postabortion care		Offer induced abortion		Offer either postabortion care or induced abortion		Offer PAC	Offer SAC	Offer either PAC or SAC
	N	N	%	N	%	N	%	N	N	%	N	%	N	%	%	%	%
All Public facilities	223	166	74.4	–	–	–	–	223	174	78.0	–	–	–	–	3.6	–	–
Facility type																	
Public Hospital	34	34	100.0	–	–	–	–	34	34	100.0	–	–	–	–	0.0	–	–
Public Health center IV	54	54	100.0	–	–	–	–	54	54	100.0	–	–	–	–	0.0	–	–
Public Health center III	73	59	80.8	–	–	–	–	73	68	93.2	–	–	–	–	12.3*	–	–
Public Health center II	62	19	30.6	–	–	–	–	62	18	29.0	–	–	–	–	− 1.6	–	–
Location^a^																	
Urban	30	21	70.0	–	–	–	–	30	24	80.0	–	–	–	–	10.0	–	–
Rural	192	145	75.5	–	–	–	–	192	149	77.6	–	–	–	–	2.1	–	–

^a^One facility in Uganda is missing an Urban/Rural designation

* *p* < 0.05

In Uganda, 78% of all public facilities surveyed reported providing PAC in 2019, compared with 74.4% in 2018 (Table 2), but this change was not statistically significant. All Hospitals and Health Center IVs provided PAC in both rounds, and there was a significant increase in provision among Health Center IIIs between 2018 and 2019 from 80.8 to 93.2% (*p* < 0.03), while provision among Health Center IIs remained low (29–31%). PAC provision increased in urban facilities to 80% in 2019, from 70% the year before (*p* = 0.37).

### Capacity to provide basic and comprehensive postabortion care services

**Fig. 1:**
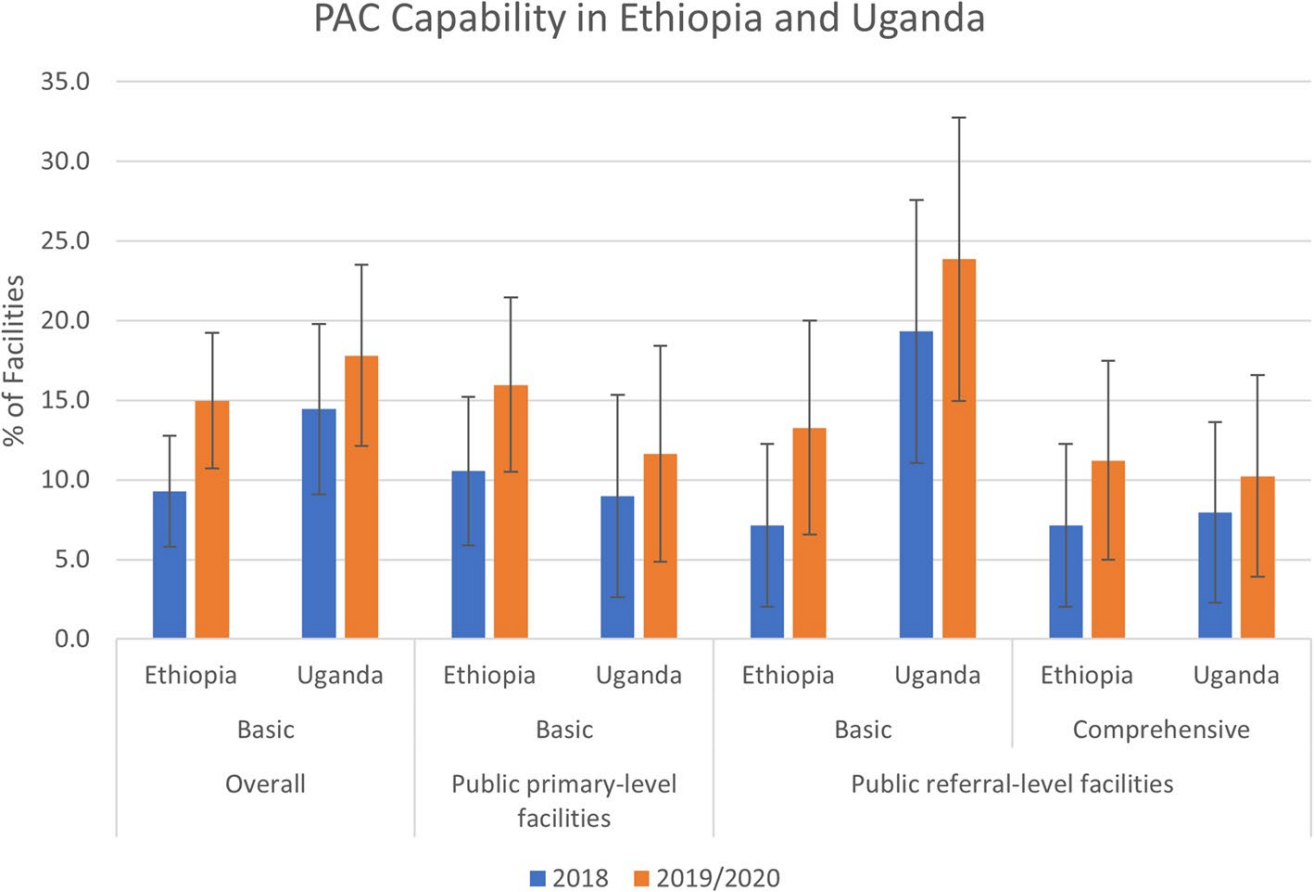
Capability of facilities to provide basic and comprehensive postabortion care services, Ethiopia 2018 & 2020 and Uganda 2018 & 2019

In Ethiopia, only 15.0% of facilities that reported providing postabortion care in 2020 had the capacity to provide all basic PAC services – 16.0% of primary-level facilities and 13.3% of referral-level facilities (Fig. 1). The proportion was lower among referral-level compared to primary-level facilities because fewer referral-level facilities met the criteria of offering family planning services 7 days per week. Among referral-level facilities, 11.2% met the requirements for providing comprehensive PAC services.

Although facilities’ capability to provide basic and comprehensive PAC was low, it improved between 2018 and 2020: overall there was an increase of 5.7 percentage points for basic PAC (*p* < 0.02) and 4.1 points among referral-level facilities for comprehensive PAC, but that change was not significant (Table 3). Improvements in PAC capability across the two surveys was largely due to the significant increase in medication abortion, which is one component in the removal of retained products (16.2 percent increase overall; *p* < 0.00). Looking at each signal function separately, between 79 and 100% of facilities provided each individual function in 2020, apart from the provision of family planning 7 days per week (16.0%), which is why PAC capability was so low.

**Table 3:** Proportion of public facilities performing individual PAC & SAC signal functions, Ethiopia 2018 & 2020

	Overall		Primary-level health facility^b^		Referral-level health facility^c^		Overall	Primary-level health facility	Referral-level health facility
	2018	2020	2018	2020	2018	2020	% change	% change	% change
Total number of facilities that provide PAC and/or SAC	262	269	164	171	98	98	–	–	–
**Capability to provide basic postabortion and safe abortion care signal functions**									
Perform removal of retained products	90.5	94.4	84.8	91.2	100.0	100.0	4.0***	6.5***	0.0
Perform manual/electric vacuum aspiration	85.5	91.1	78.7	86.0	96.9	100.0	5.6***	7.3***	3.1***
Provide medication abortion	63.7	79.9	53.0	70.2	81.6	96.9	16.2*	17.1*	15.3*
Administer parenteral antibiotics	98.9	97.8	98.2	97.1	100.0	99.0	−1.1	−1.1	−1.0
Administer uterotonics	87.4	90.3	80.5	84.8	99.0	100.0	2.9	4.3	1.0
Administer intravenous fluids	93.9	93.7	90.9	91.8	99.0	96.9	−0.2	1.0	−2.0
Provide at least one short-acting contraceptive (condoms, pills or injectables)	99.2	99.6	99.4	99.4	99.0	100.0	0.4	0.0	1.0
Facility offers family planning at least once a week	100.0	99.6	100.0	100.0	100.0	99.0	−0.4	0.0	−1.0
Facility offers family planning 7 days a week	12.2	16.0	15.2	17.5	7.1	13.3	3.8	2.3	6.1
Has 1+ staff capable of providing PAC available	100.0	100.0	100.0	100.0	100.0	100.0	0.0	0.0	0.0
Has 1+ staff capable of providing abortion available	100.0	100.0	100.0	100.0	100.0	100.0	0.0	0.0	0.0
Proportion of facilities with basic PAC capability	9.3	15.0	10.6	16.0	7.1	13.3	5.7**	5.4	6.1
Proportion of facilities with basic SAC capability	66.7	82.8	58.4	74.1	78.0	95.8	16.1*	15.7*	17.8*
**Capability to provide comprehensive postabortion and safe abortion care signal functions**^a^									
Provide dilation and evacuation	29.3	16.7	16.1	6.0	51.0	34.7	−12.7*	−10.1*	−16.3**
Provide at least one long-acting reversible contraceptive (IUDs or implants)	99.2	98.5	98.8	98.2	100.0	99.0	−0.7	−0.5	−1.0
Perform blood transfusion	39.3	37.9	6.7	5.8	93.9	93.9	−1.4	−0.9	0.0
Surgical/laparotomy capability	30.5	30.9	3.0	2.3	76.5	80.6	0.3	−0.7	4.1
Proportion of referral-level facilities with comprehensive PAC capability	–	–	–	–	7.1	11.2	–	–	4.1
Proportion of referral-level facilities with comprehensive SAC capability	–	–	–	–	38.5	32.6	–	–	−5.8

^a^Comprehensive PAC facilities must have all of the basic signal functions plus at least one long-acting reversible contraceptive method (IUDs or implants), blood transfusion and surgical capability. For comprehensive SAC, facility must also provide dilation and evacuation. Primary-level facilities are not included in the denominator for comprehensive signal functions

bPrimary-level includes Health Centers

^c^Referral-level includes Hospitals

* *p* < 0.001

** *p* < 0.05

*** *p* < 0.1

In Uganda, fewer than one in five (17.8%) facilities that reported providing PAC in 2019 had the capacity to provide all basic services – 11.6% of primary-level facilities and 23.9% of referral-level facilities. Among referral-level facilities, 10.2% met the requirements for providing comprehensive PAC services. Facilities’ capability to provide basic and comprehensive PAC improved between 2018 and 2019 in Uganda: an increase of 2.6 percentage points among primary-level facilities and 4.6 percentage points among referral-level facilities for basic PAC, and 2.2 points among referral-level facilities for comprehensive PAC (Table 4). These changes were not statistically significant. However, since this is a study of health facilities, these changes do represent real and important increases in these specific facilities. Individual services improved particularly among primary-level facilities during the time-period: the ability to remove retained products of conception increased from 21.8% in 2018 to 38.4% in 2019 (*p* < 0.02); administration of uterotonics increased from 76.9 to 90.7% (*p* < 0.02); provision of long-acting reversible contraceptive methods increased from 67.9 to 81.4% (*p* < 0.05). Blood transfusion services and capacity to provide surgery/laparotomy increased from 62.5 to 69.3% and from 63.6 to 71.6%, respectively, among referral-level facilities, but these changes were not significant. As in Ethiopia, the provision of family planning 7 days per week was low across survey rounds.

**Table 4:** Proportion of public facilities performing individual PAC signal functions, Uganda 2018 & 2019

	Overall		Primary-level health facility^b^		Referral-level health facility^c^		Overall	Primary-level health facility	Referral-level health facility
	2018	2019	2018	2019	2018	2019	% change	% change	% change
Total number of facilities that provide PAC	166	174	78	86	88	88	–	–	–
**Capability to provide basic postabortion care signal functions**									
Perform removal of retained products	52.4	59.8	21.8	38.4	79.5	80.7	7.4	16.6*	1.1
Administer parenteral antibiotics	100.0	100.0	100.0	100.0	100.0	100.0	0.0	0.0	0.0
Administer uterotonics	88.6	95.4	76.9	90.7	98.9	100.0	6.8*	13.8*	1.1
Administer intravenous fluids	91.6	94.8	83.3	89.5	98.9	100.0	3.3	6.2	1.1
Provide at least one short-acting contraceptive (condoms, pills or injectables)	97.6	98.3	96.2	96.5	98.9	100.0	0.7	0.4	1.1
Facility offers family planning 7 days a week	25.9	30.5	28.2	32.6	23.9	28.4	4.6	4.4	4.5
Has 1+ staff capable of providing PAC available	100.0	100.0	100.0	100.0	100.0	100.0	0.0	0.0	0.0
Proportion of facilities with basic PAC capability	14.5	17.8	9.0	11.6	19.3	23.9	3.4	2.7	4.5
**Capability to provide comprehensive postabortion care signal functions**^a^									
Provide at least one long-acting reversible contraceptive (IUDs or implants)	83.1	89.1	67.9	81.4	96.6	96.6	5.9	13.4*	0.0
Perform blood transfusion	34.3	35.6	2.6	1.2	62.5	69.3	1.3	−1.4	6.8
Surgical/laparotomy capability	33.7	37.4	0.0	2.3	63.6	71.6	3.6	2.3	8.0
Proportion of referral-level facilities with comprehensive PAC capability	–	–	–	–	8.0	10.2	–	–	2.3

^a^Comprehensive PAC facilities must have all of the basic signal functions plus at least one long-acting reversible contraceptive method (IUDs or implants), blood transfusion and surgical capability. Primary-level facilities are not included in the denominator for comprehensive signal functions

^b^Primary-level includes Health Centre II and Health Centre III

^c^Referral-level includes Health Centre IV and Hospitals

* *p* < 0.05

### Capacity to provide basic and comprehensive safe abortion care services

**Fig. 2:**
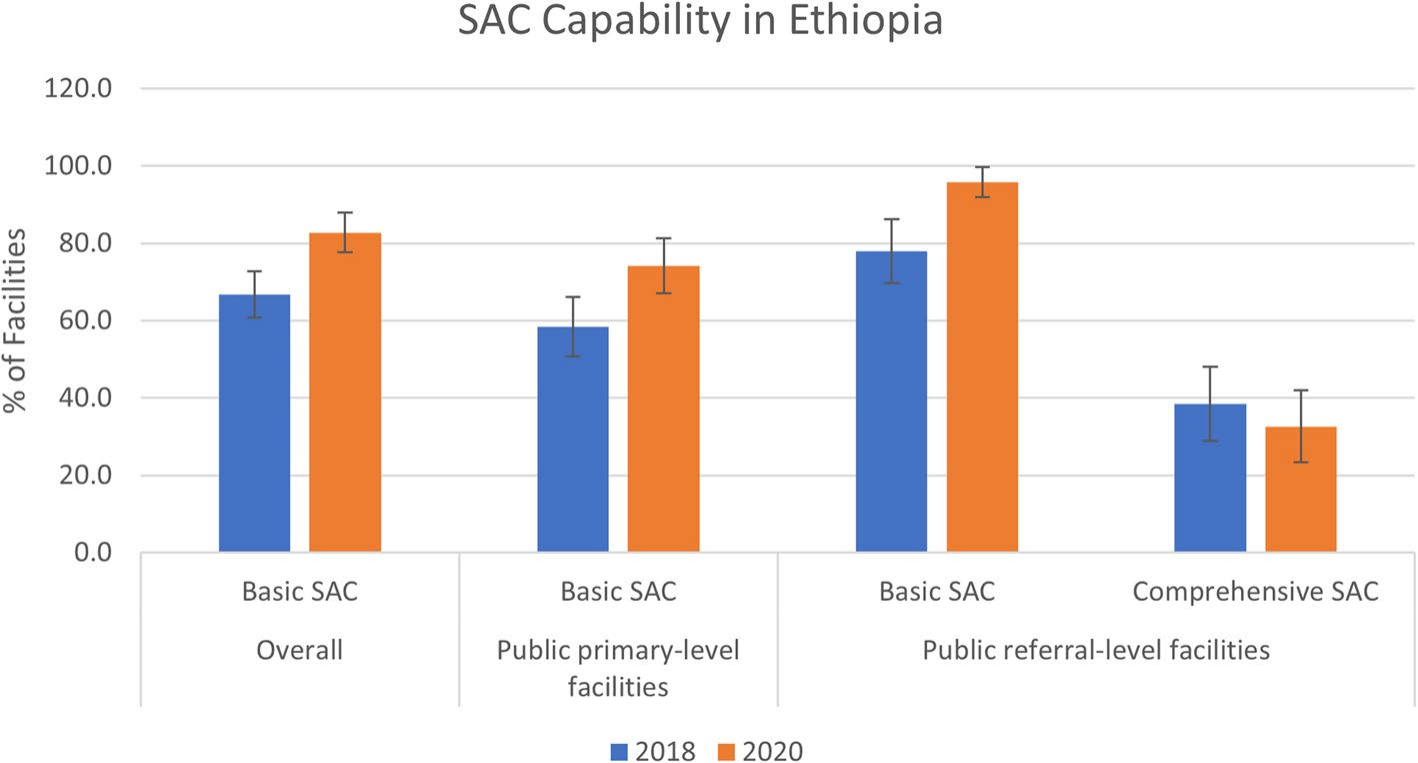
Capability of facilities to provide basic and comprehensive safe abortion care services in Ethiopia, 2018 & 2020

Overall, facilities in Ethiopia were better equipped to provide basic and comprehensive SAC services than they were to provide PAC in both rounds of surveys (Fig. 2). However, this difference is attributable to the different requirements for SAC and PAC capability – as mentioned previously, while the signal function-based classification for SAC requires that family planning be offered once per week, facilities must offer family planning 7 days per week to meet the requirements for basic or comprehensive PAC. Using these definitions, the capacity to provide all basic safe abortion services in 2020 was 82.8% overall – 74.1% of primary-level and 95.8% of referral-level facilities. Among referral-level facilities, 32.6% met the requirements for providing comprehensive abortion services.

Basic SAC increased substantially during the time-period from 66.7 to 82.8% overall (*p* < 0.00), due in large part to an increase in the provision of medication abortion (from 53.0 to 70.2%; *p* < 0.00 among primary-level facilities; 81.6 to 96.0%; *p* < 0.00 among referral-level). There was also an increase in MVA provision to a lesser extent and mostly among primary-level facilities (almost all referral-level facilities already provided MVA in 2018). However, comprehensive SAC services among referral-level facilities decreased over the 2 years, mostly due to a decrease in the proportion of facilities that provided dilation and evacuation (from 51.0 to 34.7%; *p* < 0.02).

### Postabortion and safe abortion caseloads

**Fig. 3:**
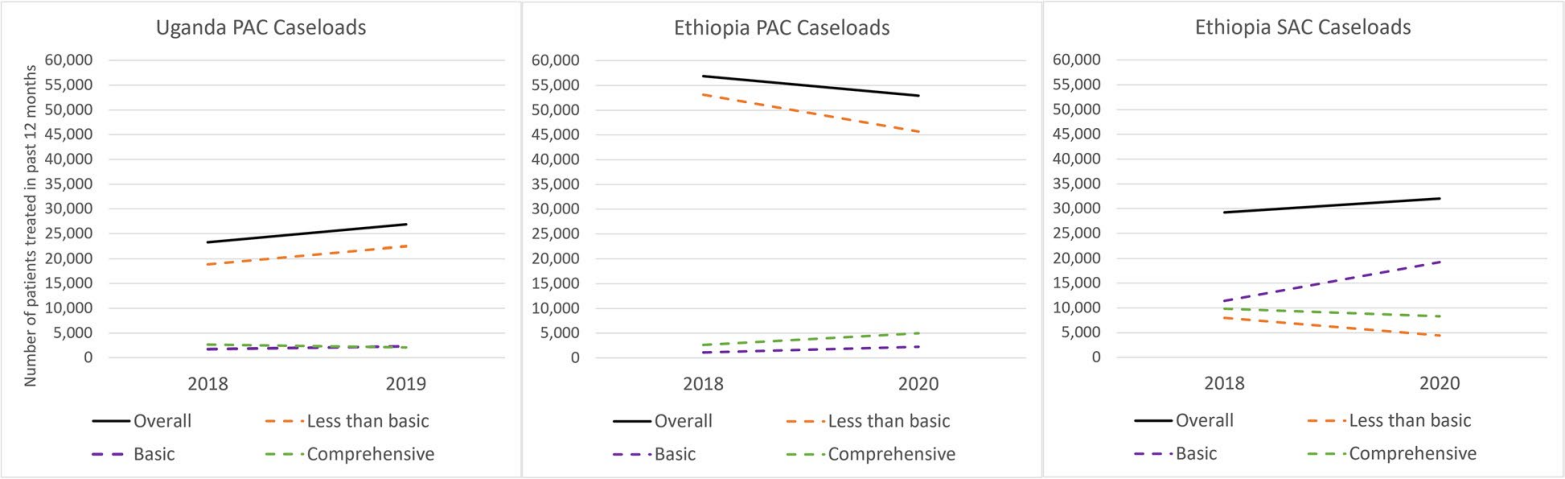
Postabortion and safe abortion care caseloads by capability in public facilities, Ethiopia 2018 & 2020 and Uganda 2018 & 2019

In Uganda, the number of PAC cases treated in facilities increased by 15.5% from 23,268 in 2018 to 26,874 in 2019 (Fig. 3). The vast majority of PAC patients were treated in facilities that were classified as having less than basic capacity for PAC (81%, *n* = 18,846 in 2018 and 84%, *n* = 22,482 in 2019). While cases also increased slightly in facilities with basic-only capability, the number of cases treated in facilities that had the capacity to provide comprehensive services decreased.

In Ethiopia, PAC cases decreased over time while SAC cases increased. There were an estimated 56,898 PAC cases treated in surveyed facilities in 2018 and 52,914 in 2020 (a 7% decrease). The majority of PAC cases were treated at facilities that were classified as having less than basic PAC capacity in both rounds, but decreased over time from 93.3% (*n* = 53,136) to 86.3% (*n* = 45,660). Cases increased slightly (from 1146 to 2232) in facilities with basic-only capability and increased (from 2,616 to 5,022) in facilities with comprehensive PAC.

There was a 9.7% increase in the estimated number of annual safe abortions in all surveyed facilities in Ethiopia, from 29,202 in 2018 to 32,022 in 2020. Among facilities that were classified as having basic-only capacity to provide abortion, the number of cases increased: from 11,430 in 2018 to 19,224 in 2020. The total number of abortions decreased over the time-period in facilities that were classified as having less than basic capability to provide SAC (from 7,962 to 4,458), and to a lesser extent in facilities with comprehensive capability (9,810 to 8,340).

There were no statistically significant changes over time in either country for the proportion of facilities providing inpatient PAC services, receiving or referring PAC patients from or to other facilities, and treating PAC patients with severe complications. Nonetheless, we present these indicators across the two rounds of surveys because they represent a real increase in PAC service provision in the facilities included in our study. All facilities with at least basic PAC capacity in Uganda reported providing inpatient care in 2018, but in 2019 this was somewhat lower at 95% among basic-only and 90% among comprehensive facilities (Table 5). The proportion that received PAC patients referred from other places was also lower in 2019 compared with 2018, especially among facilities with only basic capability (33.3% vs. 57.1%). The proportion referring PAC cases elsewhere was relatively constant, but among facilities with comprehensive PAC 14.3% referred in 2018 and none were referring patients 1 year later. Severe complications were slightly lower in 2019 overall (10.3% compared to 8.2%).

**Table 5:** Postabortion care caseloads by PAC capability in public facilities, Ethiopia 2018 and 2020 and Uganda 2018 and 2019

	Ethiopia											
	2018				2020				% change 2018–2020			
		PAC Capability				PAC Capability				PAC Capability		
	Overall	Less than basic	Basic	Comprehensive	Overall	Less than basic	Basic	Comprehensive	Overall	Less than basic	Basic	Comprehensive
Total number of facilities that provided PAC	259	235	16	8	267	227	29	11	–	–	–	–
Total number of PAC patients treated in the past 12 months^a^	56,898	53,136	1,146	2,616	52,914	45,660	2,232	5,022	–	–	–	–
Average number of PAC patients treated in the last 12 months per facility^a^	220.5	227.1	71.6	327	198.2	201.1	77.0	457	–	–	–	–
	%	%	%	%	%	%	%	%	%	%	%	%
Facilities that treated any PAC cases as inpatients in the past month	78.3	77.7	75.0	100.0	71.3	71.8	55.6	100.0	−6.9*	−5.9	−19.4	0.0
Facilities that treated PAC cases referred from elsewhere in the past month	35.6	37.0	7.1	50.0	37.0	37.2	21.7	63.6	1.4	0.2	14.6	13.6
Facilities that referred PAC cases elsewhere in the past month	21.0	20.8	21.4	25.0	15.0	14.5	17.4	18.2	−6.0*	−6.3*	−4.0	− 6.8
Facilities that treated PAC patients with severe complications^b^ in the past month	11.1	11.3	0.0	25.0	8.1	8.0	0.0	27.3	−3.0	−3.3	0.0	2.3
	**Uganda**											
	2018				2019				% change 2018–2019			
		PAC Capability				PAC Capability				PAC Capability		
	Overall	Less than basic	Basic	Comprehensive	Overall	Less than basic	Basic	Comprehensive	Overall	Less than basic	Basic	Comprehensive
Total number of facilities that provided PAC	166	142	17	7	174	143	21	10	–	–	–	–
Total number of PAC patients treated in the past 12 months^a^	23,268	18,846	1,752	2,670	26,874	22,482	2,304	2,088	–	–	–	–
Average number of PAC patients treated in the last 12 months per facility^a^	147.3	139.6	109.5	381.4	155.3	158.3	109.7	208.8	–	–	–	–
	%	%	%	%	%	%	%	%	%	%	%	%
Facilities that treated any PAC cases as inpatients in the past month	89.0	87.1	100.0	100.0	86.3	84.8	95.0	90.0	−2.7	−2.3	−5.0	−10.0
Facilities that treated PAC cases referred from elsewhere in the past month	48.9	47.4	57.1	57.1	39.9	40.0	33.3	60.0	−9.0	−7.4	−23.8	2.9
Facilities that referred PAC cases elsewhere in the past month	29.4	29.3	38.5	14.3	27.3	27.4	36.8	0.0	−2.1	−1.9	−1.6	−14.3
Facilities that treated PAC patients with severe complications^b^ in the past month	10.3	8.7	14.3	28.6	8.2	5.8	15.8	28.6	−2.1	− 2.9	1.5	0.0

^a^Among all facilities that reported providing PAC in either round

^b^Complications that included a perforated uterus or gut requiring laparotomy, intensive care unit admission, or organ failure

* *p* < 0.1

In Ethiopia, compared to in 2018, fewer facilities overall reported treating inpatients in 2020 (71.3%, compared to 78.3%). All facilities with comprehensive PAC capability treated inpatients in both survey rounds, but only 55.6% of facilities with basic-only capability treated inpatients in 2020 compared with 75% in 2018. More facilities with basic and comprehensive PAC capacity received PAC patients referred from elsewhere in 2020 compared with 2018: 21.7% vs. 7.1% for basic PAC facilities and 63.6% vs. 50.0% with comprehensive PAC. Referrals of PAC patients to a different facility was not reported as commonly as it was in 2018 among facilities with all levels of capacity. Similar to in Uganda, severe complications were slightly lower in 2020 overall (11.1% compared to 8.1%).

There were significant decreases in the proportion of facilities in Ethiopia that reported receiving any abortion clients referred from elsewhere between 2018 and 2020: from 30.5 to 19.7% (*p* < 0.00; Table 6).

**Table 6:** Safe abortion care in public facilities, Ethiopia 2018 and 2020

	All Facilities		
	2018	2020	% change 2018–2020
Total number of facilities that provided abortions	216	238	–
Total number of induced abortion clients in the past 12 months^a^	29,202	32,022	–
Average number of induced abortion clients in the past 12 months per facility^a^	135.2	134.5	–
Total number of induced abortion clients in the past 12 months^b^	28,338	28,296	–
Average number of induced abortion clients in the past 12 months per facility^b^	138.9	138.7	–
	%	%	%
Facilities that provided second trimester abortions in the past month	36.4	38.1	1.7
Facilities receiving any abortion clients referred from elsewhere	30.5	19.7	−10.8*

^a^Among all facilities that reported providing safe abortion care in either round

^b^Only among the 216 facilities that reported providing safe abortion care in both rounds

* *p* < 0.001

## Discussion

Despite previous concerns that facilities’ capacity to provide PAC, and especially to provide SAC, may be negatively affected by the GGR, the findings from this analysis suggest that PAC service provision in both countries, and SAC provision in Ethiopia, either remained consistent or even improved during the time-period when the GGR was in effect. In Ethiopia, approximately 95% of public facilities reported that they provided any PAC services, which is consistent with other data from the region, [38, 39] and this proportion stayed fairly constant between 2018 and 2020. PAC service provision was lower in facilities in Uganda but still widespread. The proportion of facilities providing PAC increased slightly between 2018 and 2019, however this overall increase was primarily driven by higher level facilities. In line with previous trends in Ethiopia, [17] the proportion of facilities providing SAC in our sample significantly increased from 76.6 to 84.4%, with the biggest increases found among lower-level facilities (health centers) and those in rural areas.

While a majority of facilities provided PAC (and SAC in Ethiopia), the findings show that their capacity to provide basic or comprehensive services was low overall. However, there is no evidence to suggest that facilities’ capacity to provide care declined while the GGR was in effect. In fact, the provision of most individual services was relatively high, and improved over time, in both countries. Capacity to provide safe abortion services was higher and increased during the time-period; however comprehensive SAC capability at referral-level facilities decreased due to a decrease in D&E service availability, which could be reflective of the chill effect of the policy. Improvements in safe abortion services over time were largely attributable to a widespread increase in the availability of medication abortion, and to some extent MVA, in facilities. In Uganda, the capacity to provide basic and comprehensive PAC was slightly higher than was observed in Ethiopia, and also improved over the time-period among both primary-level and referral-level facilities that provided PAC. Individual services provided by primary-level facilities in particular improved over time, most notably in their ability to remove retained products, administer uterotonics and to provide long-acting reversible contraceptive methods.

**Fig. 4:**
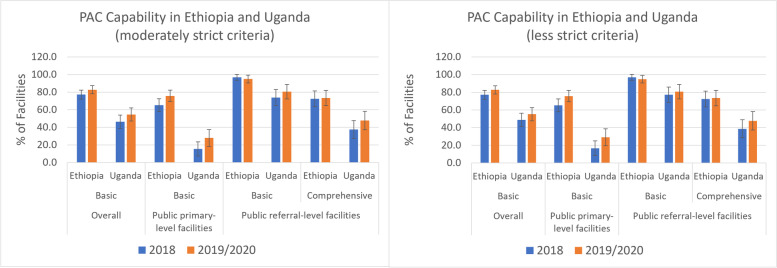
Capability of facilities to provide basic and comprehensive PAC services using a moderately strict and less strict criteria, Ethiopia 2018 & 2020 and Uganda 2018 & 2019

The signal function for PAC that was least likely to have been met in both countries is the availability of family planning services 7 days per week. Despite improvements from 2018, few facilities in Ethiopia provided these services every day in 2020 (17.5% of primary-level and 13.3% of referral-level facilities; Table 3), which brought down the overall proportion of facilities classified as providing basic and comprehensive PAC. A similar pattern was observed in Uganda: the availability of family planning services was higher than in 2018 but still not commonly available every day in 2019 (32.6% of primary-level and 28.4% of referral-level facilities reported providing family planning 7 days per week; Table 4). Family planning provision is a fundamental element of PAC/SAC service delivery, and small changes to this signal function have large impacts on facilities’ overall capacity to provide PAC and/or SAC. For example, requiring family planning services only 5 days per week instead of 7 would increase the proportion capable of providing basic PAC in Ethiopia from 15.0 to 82.8% overall (75.7% among primary-level and 94.9% among referral-level) and the proportion providing comprehensive PAC services from 11.2 to 73.5% among referral-level facilities (Fig. 4). In Uganda, the proportion capable of providing basic PAC would increase from 17.8 to 54.6% overall (27.9% among primary-level and 80.7% among referral-level) and the proportion providing comprehensive PAC services would increase from 10.2% using the strictest criteria to 47.7% among referral-level facilities.

However, this is not a call to weaken the criteria for the family planning signal function. That would also mean that some women would not be able to access postabortion family planning if they came on the days that family planning was unavailable. Rather, we suggest that using the number of days per week that family planning is available to capture the availability of postabortion family planning is flawed because it may not reflect the services which are actually being offered at the time of the abortion/postabortion service unless those services are integrated. A more appropriate signal function would be the proportion of facilities that offer family planning as a routine part of the abortion or postabortion services, at the same time and place as the service, ideally by the same provider. Capturing the integration of these services would allow for a much more accurate estimation of facilities’ capacity.

Despite fears that the GGR might impact the safety of abortion in Ethiopia, our results suggest an opposite trend during this time period. Overall, we observed a 9.7% increase in safe abortions in surveyed facilities in Ethiopia. While some of this increase could be due to population growth during that time period, the population of women aged 15–49 only increased by 6.7% from 2018 to 2020, and likely does not account for all of the observed increases in this study [35]. At the same time, fewer women were treated for postabortion complications in facilities. Increasing facility abortions should decrease overall complications and therefore decrease the need for PAC in the population, so this trend is consistent. Further, the increase in the proportion of facilities capable of providing basic and comprehensive SAC services in conjunction with the increase in the annual number of safe abortions reported over this time-period suggests that access to abortion care has improved. As such, the reduction in facilities treating PAC inpatients was likely due to the decrease in patients presenting with severe complications, as has been documented elsewhere, [39] rather than to a lack of capacity among facilities.

There was also a shift in care seeking for postabortion complications from facilities with less than basic capacity to provide PAC in 2018 to those with basic or comprehensive capacity in 2020, indicating that more women are getting services at better equipped facilities. Further, the decrease in the proportion of facilities that referred PAC patients to other facilities could indicate that referral systems were disrupted; it is more likely, however, especially since we observed an increase in the proportion treating PAC patients referred from elsewhere, that lower-level facilities expanded their capacity to provide services and thus did not have to refer patients elsewhere as often.

In contrast to Ethiopia, evidence from this study suggests that the GGR may have resulted in negative impacts on abortion care and/or safety in Uganda. While the population of women aged 15–49 was estimated to have increased by 8% from 2018 to 2019, [40] we observed a 15.5% increase in the number of PAC cases during this period. Some of this increase in public-sector cases may represent a shift in where women are accessing care within the health system rather than an increase in the number of complications occurring, since private facilities were more likely to be directly impacted by the GGR policy. Nevertheless, it is plausible that some proportion of the increase is a true increase in the need for PAC and could be an indication that abortion became less accessible, potentially more stigmatized, and therefore less safe during the period in which the GGR was in effect.

While we observed improvements in facilities’ capacity to provide basic and comprehensive services in Uganda, the majority of PAC cases were treated in less-equipped facilities (less than basic capability), and the number receiving treatment at facilities equipped to provide comprehensive services decreased over time. This change could be because women used safer methods of abortion, including misoprostol which is increasingly available and critical in settings where abortion is legally restrictive, and did not experience severe complications for which they would need care at the highest level facilities. However, it could also be indicative of disruptions in referral mechanisms resulting in a lack of access to care at the most equipped facilities. In Uganda, the proportion of facilities that treated PAC patients referred from elsewhere decreased over time, especially in facilities with basic-only capability (57.1% in 2018 to 33.3% in 2019). Moreover, referrals of PAC patients *out* to other providers decreased over time among all facility types surveyed, especially those with comprehensive PAC capacity (from 14.3 to 0.0%). Given that we know the GGR prohibits NGOs from either serving or referring for abortion services, these decreases could be indicative of a chilling effect and overinterpretation of the policy.

### Limitations

There are several limitations of our study. This analysis looked at changes during the period in which the GGR was in effect, but it is possible that other, unmeasured, factors affecting supply and demand of services were occurring over this period as well. Therefore, we cannot make causal inferences related to the impact of the GGR but rather present a descriptive account of what happened in these countries during a period of time. Further. a stronger study design would have included more data from the years prior to the GGR taking effect, which would better help establish trends in key outcomes prior to the GGR’s implementation.

Another limitation of this study is its lack of generalizability. First, this study relied on building off of preexisting PMA survey platforms in Ethiopia and Uganda, which were not designed to sample private facilities capable of providing PAC, and our analyses are only conducted among public facilities. However, it is almost certain that private facilities were differentially impacted by the GGR, as the main target of the policy was on US government support to non-US NGOs operating in each country. This means that we cannot present the full picture of the policy’s impact on services in both countries. However, our analyses still provide valuable insight into the impact of the GGR in Ethiopia and Uganda. First, even though the policy did not target public facilities, previous research has shown that the GGR did have an impact on service delivery in public sector facilities, largely driven by the fact that NGOs provide technical support and assistance to the public health system in both countries [7, 9]. Further, while the primary target of the policy was non-US NGOs, the hypothesized downstream effects of the policy (ie. increases in unintended pregnancy and abortion, shifts between public/private service utilization) should be detectable within the larger public health system. Finally, the samples were not nationally representative of all public facilities in each country, further limiting generalizability. The findings are probably a close approximation in Uganda, but the sample is missing key regions in Ethiopia (although it did capture the most populous areas).

Other signal functions papers have attempted to assess the quality of care provided in facilities. This paper only assesses structural capacity at the health-system level, which is just one of several important aspects to consider when measuring quality of care. While we were able to present the availability of essential services and document critical strengths and gaps in services, we were not able to capture how those services were delivered and experienced by the women receiving them, thus we cannot draw conclusions about the quality of the care provided.

Previous signal functions work, where possible, also distinguished between facilities’ capacity to provide abortion services in the first and second trimester. We did not have specific questions to capture these differences. While capacity increased overall, the declines in capacity among referral-level facilities, particularly regarding D&E in Ethiopia, could indicate a reduction in the availability of second trimester abortion services that we are not fully capturing in our data and analysis.

As mentioned earlier, the availability of family planning is a crucial indicator to which the overall capacity scores were quite sensitive. Relaxing the requirement for PAC from 7 days to 5 days resulted in a significant increase in the proportion of facilities that could provide services in both countries. We considered 1 day a week for family planning availability to be sufficient for abortion care under the assumptions that abortion services are not usually provided on an emergency basis and that, in most cases, appointments can be scheduled around the day when family planning is available. However, it may be the case that abortion services are not predominantly scheduled ahead of time; future studies should investigate to what extent that assumption is typically met. Moreover, using the number of days per week family planning is provided as a proxy for the availability of family planning at the time of the service is problematic. For example, just because family planning services are available everyday does not necessarily mean that facilities are providing it as part of the constellation of abortion care. For the family planning signal function to be more meaningful, it should be linked to the abortion/postabortion services in question. To more accurately describe service integration, researchers should consider asking whether or not family planning is routinely provided as part of the abortion or postabortion service, provided at the same time and in the same room, potentially by the same provider.

It is also possible that population increases may be driving some increases in abortion caseloads. While we tried to remedy this by looking at the estimated percent increase in the populations of women of reproductive age in each country over the study period, we cannot fully disentangle what increases are due to changing patterns in abortions, population growth, or an increase in pregnancies.

Finally, the lack of data on abortion service provision in Uganda is a limitation in our study. Due to the restrictive laws and stigma surrounding abortion in the country, the study team decided not to include the induced abortion module in Uganda. Abortions do occur in restrictive contexts, both within and outside the bounds of the law, and maintaining the availability of those services is critical. While abortion service provision in restrictive settings is likely even more vulnerable to changes due to the policy, and is important to explore, we did not want to draw attention to these services and inadvertently disrupt them.

## Conclusions

This is one of the only studies to use signal functions to document changes to facilities’ capacity to provide services across two points in time. This is a useful approach that could be adapted by others to assess facility capacity to provide PAC and/or SAC, or other sexual and reproductive health services, over time. The findings from this analysis suggest that the level of PAC services available in Uganda and PAC and SAC services in Ethiopia did not significantly change during the Global Gag Rule time-period. We had hypothesized that there would have been a reduction in the availability of supplies needed for safe abortion as well as postabortion care services in facilities due to the chilling effect of the GGR, but it appears individual services within facilities continued to improve over time, which is consistent with the overall trend documented prior to the policy [17]. The increase in PAC cases observed only in Uganda could be an indication of several underlying trends, including an increase in the total number of abortions, a decrease in the proportion of facility-based abortions and an increase in the proportion of abortions that are unsafe, or a combination of the two. The extent to which this is the case, and whether any trends are associated with the GGR, requires further investigation.

Despite improvements overall among primary-level facilities, the low levels of D&E services and their reduction over time especially in higher-level facilities in Ethiopia must be addressed. Women who need second trimester abortion services are among the most vulnerable to negative health outcomes – later term abortions account for a small proportion of induced abortions overall, but are associated with more severe complications than first trimester abortions [41]. Thus it is critical to ensure that women have access to safe services in the second trimester. D&E has been proven safe and effective for second trimester abortion given the availability of proper equipment and trained providers, and in circumstances where medication abortion or MVA may not be appropriate or desired. However, D&E has not been established as a widely used method in Ethiopia, and our findings show that only half of referral-level facilities reported providing the service in 2018, reduced to 35% 2 years later. The extent to which this reduction is reflective of a direct impact of the policy, or just a demonstration of the already established low availability of the method, should be explored further. Regardless, steps should be taken to fill this gap in provision: abortion providers should undergo clinical training and facilities must have adequate supplies to ensure that D&E can be provided safely.

This analysis also highlights a need for improving the availability of family planning services and integrating them into both PAC and SAC services at all levels of facilities. Since most people having an abortion or receiving postabortion care do not want to become pregnant in the near future, postabortion family planning is an essential part of this constellation of care. Ensuring that family planning is offered at the time of the service, in the same room and ideally by the same provider, will greatly improve facilities’ capacity to provide basic and comprehensive care.

## Data Availability

The datasets generated and/or analyzed during the current study (in 2018 and 2019) are public and available by request here: https://www.pmadata.org/data/available-datasets. The Ethiopia data from 2020 is available from the corresponding author on reasonable request.

## Abbreviations

EA: Enumeration area
EmOC: Emergency obstetric care
EVA: Electric Vacuum Aspiration
GGR: Global Gag Rule
IUD: Intra-uterine device
MVA: Manual Vacuum Aspiration
ODK: Open Data Kit
PAC: Postabortion care
PMA: Performance Monitoring for Action
SAC: Safe abortion care
