# Correction: Postabortion and safe abortion care coverage, capacity, and caseloads during the global gag rule policy period in Ethiopia and Uganda

**DOI:** 10.1186/s12913-023-09252-7

**Published:** 2023-03-10

**Authors:** Melissa Stillman, Simon P. S. Kibira, Solomon Shiferaw, Fredrick Makumbi, Assefa Seme, Elizabeth A. Sully, Lilian Ha, Margaret Giorgio

**Affiliations:** 1 Guttmacher Institute, 125 Maiden Lane, 10038 New York, NY USA; 2 School of Public Health, College of Health Sciences, Makerere University, New Mulago Hill Rd, Kampala, Uganda; 3 School of Public Health, Addis Ababa University, Addis Ababa, Ethiopia

**Correction to**: BMC Health Services Research (2023) 23:104. 10.1186/s12913-022-09017-8.

Following publication of the original article [1], the authors identified an error in the author name of Lilian Ha.

The incorrect author name is: Lilian Han.

The correct author name is: Lilian Ha.

The author group has been updated above and the original article [1] has been corrected.

## References

[1] Stillman et al. Postabortion and safe abortion care coverage, capacity, and caseloads during the global gag rule policy period in Ethiopia and Uganda. BMC Health Serv Res. 2023;23:104. 10.1186/s12913-022-09017-8 10.1186/s12913-022-09017-8 PMC9890752 36726121

